# Development of a Simulation Model for Digital Twin of an Oscillating Water Column Wave Power Generator Structure with Ocean Environmental Effect

**DOI:** 10.3390/s23239472

**Published:** 2023-11-28

**Authors:** Byungmo Kim, Jaewon Oh, Cheonhong Min

**Affiliations:** 1 Ship and Offshore Technology Team, Korean Register, 36 Myeongji Ocean City 9-ro, Gangseo-gu, Busan 46762, Republic of Korea; bmkim@krs.co.kr; 2 Offshore Industries R&BD Center, Korea Research Institute of Ships & Ocean Engineering, 1350 Geojebuk-ro, Jangmok-Myeon, Geoje-si 53201, Gyeongsangnam-do, Republic of Korea; herotaker@kriso.re.kr

**Keywords:** offshore structure, wave power generator, digital twin, added mass, environmental effect, modal analysis

## Abstract

This research article focuses on developing a baseline digital twin model for a wave power generator structure located in Yongsu-ri, Jeju-do, South Korea. First, this study performs a cause analysis on the discrepancy of the dynamic properties from the real structure and an existing simulation model and finds the necessity of modeling the non-structural masses and the environmental factors. The large amounts of the ballast are modeled in the finite element model to enhance the accuracy of the digital twin. Considering the influence of environmental factors such as tide level and wave direction, the added mass effect of structural members, one of the hydrodynamic effects, depending on the change of the ocean environments is calculated based on the rule of Det Norske Veritas and applied. The results indicate that non-structural mass components significantly impact the dynamic characteristics of the structure. Additionally, environmental factors have a greater effect on the dynamic behavior of the box-type structure compared to lightweight offshore structures.

## 1. Introduction

Global warming attributed to the greenhouse gases, such as carbon dioxide and methane, is the biggest hurdle for every living entity around the world, as well as just human beings. To replace carbon-based energy resources, i.e., petroleum and coal which emit greenhouse gases, a variety of alternative energy resources have been developed. However, it is required for them to be eco-friendly, renewable, and sustainable. The power of winds, waves, tides, and currents in the ocean is a representative example of satisfying the requirements.

**Figure 1:**
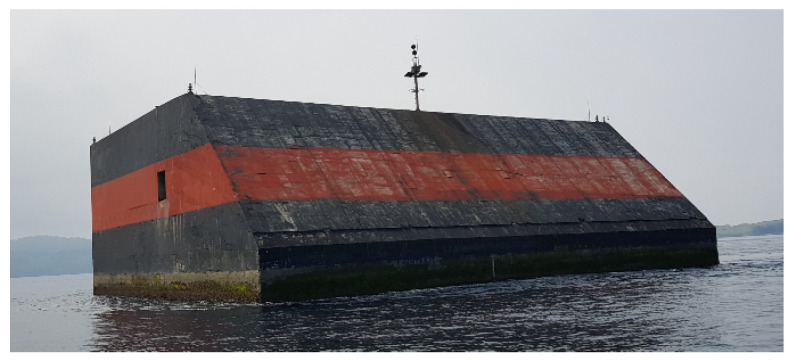
OWC-type Yongsu wave power generator [1].

Figure 1 [1] shows the structure of Yongsu wave power generator, which is the prototype for the real sea experiment located on the coastal waters of Jeju Island in South Korea. As seen in the figure, it is a fixed-type concrete box structure with an open chamber room. The electric power generating system is the oscillating water column (OWC) type, which converts the upward and downward change of water level in the open chamber into the electric resource. To be more specific, the sea waves induce the water surface level in the chamber room up and down. The repeated movement of the water level acts as a piston pushing the air in the room, and in turn, the pressure makes the air flow into the generator.

**Figure 2:**
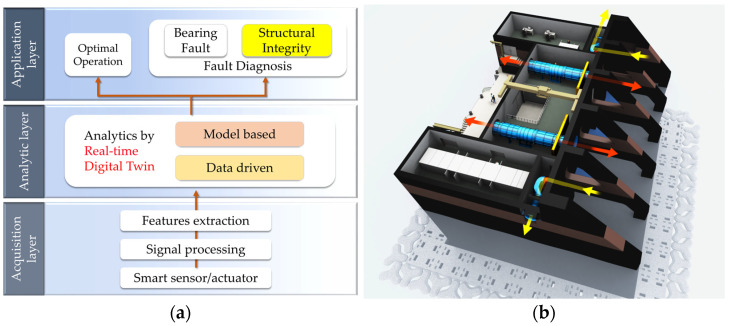
(**a**) Developing outline; (**b**) a digital twin of the wave power generator.

Korea Research Institute of Ships and Ocean Engineering (KRISO) has performed a research project to develop a digital twin. Figure 2 shows the developing outline and the concept of the digital twin. The research has been conducted in two aspects. One is related to the operation of this wave power generator system, and another is for maintenance. As for the former, Seo et al. [2] performed the study to improve wave power plant (WPP) efficiency through predictive techniques for power generation and failure diagnosis. First, correlating data from the oscillating water column (OWC) chamber was investigated through the KNIME machine learning framework, a big data analysis system. An optimal training model for predicting wave energy converter (WEC) states was developed using a digital twin of an operational OWC–WEC. Results, including those from a CNN-LSTM-based model, confirmed the effectiveness of the proposed predictive model. As for the latter, on the other hand, two maintenance points exist, the faults of the generator bearing and structural integrity. Kim et al. [3] explores fault diagnosis of the bearing in the wave power generator for maximum efficiency. Beginning with FMEA analysis to identify failure modes, the study focuses on reproducing and monitoring thrust-bearing faults using three machine learning algorithms—naïve Bayes, k-nearest neighbor, and multi-layer perceptron. The research suggests that this approach provides an efficient fault diagnosis method for wave power systems.

**Figure 3:**
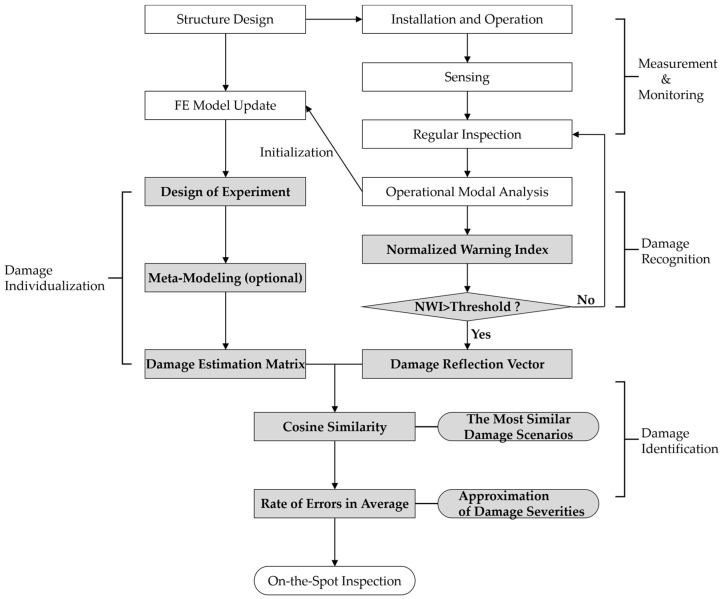
Entire flowchart of cosine similarity-based SHM [4].

As the last part of the digital twin, the first study was performed by Kim et al. [4] to develop a fast and effective technique and procedure based on cosine similarity with sensor data to enhance real-time structural health monitoring for offshore structures. The proposed method introduces the rate of changes in measured natural frequencies as a main parameter. As shown in Figure 3, it provides a normalized warning index to alert to damage occurrences, and the parameter is compared to those values from a simulation model in different damage scenarios, simplifying damage identification. As a result, the method contributed to improved usability in the field. In the second study [5], Kim et al. found that how precisely the simulation model represents the dynamic properties of a real structure is very important in applying the proposed method to the structure. They came to the conclusion that the accuracy of the simulation model should comprise external factors, such as the ocean environments on site, in the structural model itself, which affect the change of the dynamic properties of the structure, ensuring the high accuracy of the proposed technique.

For this reason, as the third research on this digital twin of the structure, this study conducts a cause analysis of the disagreement with the simulation model and this structure and improves the simulation model to be a structural baseline digital twin model. In particular, the non-structural mass components and environmental factors are dealt with.

### 1.1. Structural Issue under Uncertainty of Wave Power Generator

**Figure 4:**
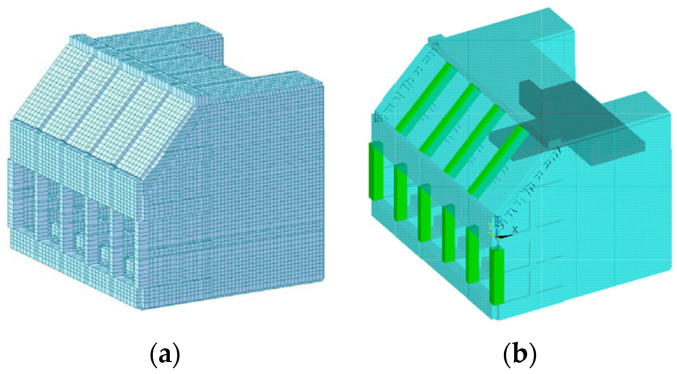
Simulation model of the Yongsu wave power generator structure: (**a**) premade model; (**b**) FE model in this study.

As with many other renewable energy systems which directly harness natural phenomena, uncertainty with suddenness was found during the test operation of the system. To explain, the oscillating water column in the chamber sometimes rises too high due to a big wave like a swell or accidental superposition of waves. It causes electrical faults, and therefore, it was considered to add a device in the air hole connecting the chamber to the generator. The basic concept of the new system is to prevent the power generator from excessively producing electric power by operating the device to block the air hole to stop the strong airflow. However, this approach caused another issue. The high pressure due to the air that is strongly pressed by the water below but cannot flow out in the blocked chamber room exerts force on the concrete structure of the room. For this matter, the code check based on the simulation model was carried out [6]. The model seen in Figure 4a was made by MIDAS CIVIL which is a commercial structural analysis software, and the internal forces of the structural members were evaluated under three load cases that include internal air pressure of 78.48, 90.0, and 110.0 kPa in the chamber. Based on the internal force of the structure members, the code check was performed to estimate the structural safety parameters of reinforcement concrete, i.e., the bending strength, the ratio of reinforcements, and rebar spacing. As a result, the risk of concrete failure was reported [6].

### 1.2. Digital Twin System of General Structures

To overcome this problem caused by uncertainty, a digital twin system that precisely monitors the state of the power generator and structure was paid attention to in this study. The concept of digital twins has been proposed in various industries for several recent years since it initially arose almost 20 years ago. Many research groups and companies have defined and utilized them as suitable methods for their purpose, such as a digital twin building [7], a digital twin factory [8], or a digital twin city [9]. A comprehensive summary of the various concepts and technical applications was carried out in several review articles [10,11]. The fundamental concept in common is that the thing identical to the structure in reality is established as the cyber system, and then linked to the physical system so that they communicate and work concurrently in real time. This is the reason this study focused on the digital twin system of this structure.

Meanwhile, it is required to accurately copy reality to develop the digital twin. There are several research articles that tried such a purpose. Several studies focused on architecture on land, such as bridges. For instance, Shim et al. [12] have proposed a framework for bridge maintenance by employing the concept of a 3D digital twin model. In their approach, real images captured using a 3D scanner during inspections are processed to identify surface cracks and internal strand cable corrosions. Subsequently, this information is integrated into a 3D building information model (BIM). Interactively, the information is accumulated in the analysis model, as well. This study presents a clear and well-defined concept of a digital twin of bridge structures, effectively bridging the gap between real-world information, the 3D BIM model, and the simulation model. However, it is worth noting that certain types of implicit damage, which may not be readily observable using a 3D scanner, pose challenges for detection in this methodology. Kang et al. [13] presented an alternative approach to overcome the aforementioned limitations by introducing a health monitoring method, with a focus on natural frequency as a key parameter of structural dynamic characteristics. In their study, the modulus of elasticity was employed as an identification parameter, and model updating techniques were employed to determine the optimal value of this parameter, ensuring that the simulation model accurately replicated the measured natural frequency. Furthermore, the authors generated input scenarios for variables such as temperature, force, wind speed, and more, utilizing the design of experiments technique. Structural responses of the updated model under these varied conditions were calculated and accumulated, and then, the data were used to train artificial neural networks, enabling them to learn and model the structural responses effectively.

### 1.3. Challenges and Cause Analysis in Developing a Digital Twin of Offshore Structures

In the offshore structure field, since a portion of offshore structures remains submerged, efficient and novel sensing utilities like 3D scanners are not easily applicable, and the areas where sensors can be attached are significantly restricted. Yi et al. [14] installed accelerometers on the Gageocho Ocean Research Station, a jacket-type offshore structure in Korea, above sea level, and conducted an analysis using data from sensors. They examined the dynamic properties of the structure, such as its natural frequencies and mode shapes. Subsequently, Kim and Yi [15] developed a precise finite element model for the structure by applying a model updating technique, accounting for mass reallocation and material properties. To be specific, they pointed out the significant difference between the measured natural frequencies and the natural frequencies from the initial FE model, performed the cause analysis for that matter, and stated that there are several usual causes of the offshore structure FE model, such as the ignored non-structural masses (appurtenance, equipment, and facilities) and material uncertainty of the concrete filled in the jacket legs. By achieving accurate model updating, they found that the strength of the concrete filled in the legs is highly likely to be much less than the design target, and emphasized the importance of considering the mass effect. Meanwhile, Kim et al. [5] investigated the suitability of a similarity-based structural health monitoring method for this offshore structure. They identified the variation of natural frequency induced by nonstructural effects, such as changes in oceanic environmental conditions, as a major challenge in monitoring structural integrity. In detail, it is worth noting that while temperature and wind speeds are the primary environmental factors affecting land architectures, offshore structures are complicatedly influenced by a more complex set of environmental factors, including tides and waves.

To summarize, several causes induce the difference between a simulation model and a real structure, and they are the major challenges for a baseline digital twin of an offshore structure. Both a simulation model and an actual architecture are established, respectively, based on the design drawings. To analyze the causes of the disparities between the simulation model and the real structure, therefore, it is helpful to compare each of them to the design drawings rather than to compare each other. This study briefly investigates the usual causes of the disparity in the aspect of modeling and reality compared to the design drawings in Table 1. Some of the causes remain virtually constant, and therefore, additional or advanced modeling with initial tuning is required. Others fluctuating over time need to be parametrically modeled as well as the relation between these causes, and the inducer of the fluctuation, i.e., environmental factors, should be identified.

Regarding this structure, the non-structural mass should be predominantly considered. The reason for this is that most of the internal spaces of the structure were filled with ballast, which was ignored in [6]. Other typical causes in general structures should be accurately identified through model tuning or model updating by comparison to measurements. For example, refs. [14,15] coped with the jacket-type offshore structure on site to update the uncertain material properties using a simulation model and the ambient sensor data. Yi et al. [16] performed an impact test on a real breakwater caisson using a ship collision and verified the structural uncertainty by comparing the acquired data to the simulation model.

In addition, factors that vary over time must be essentially modeled for the digital twin model, and hydrodynamic effects are the most significant among them. This is because this box-type structure, unlike a steel jacket-type offshore structure, has a large displacement volume in water compared to its size, and biofouling and corrosion on the surface of a concrete structure are less predominant. While the mass of the ballast remains consistent after construction, hydrodynamic effects vary depending on environmental fluctuations, such as tides and waves.

### 1.4. Contribution and Flowchart in Developing the Digital Twin Baseline Model

**Figure 5:**
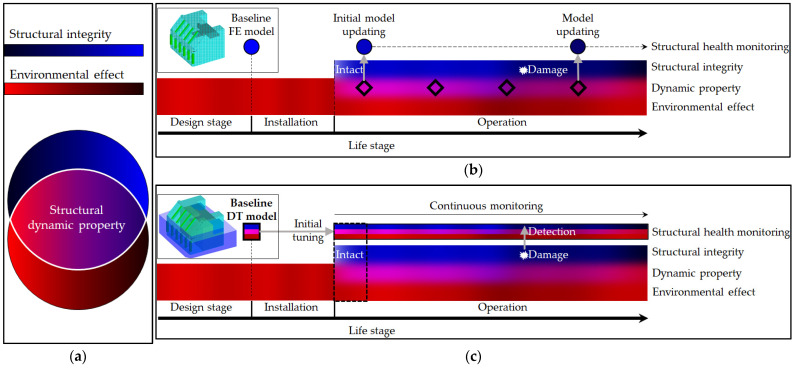
Concept and feature of FE-based digital twin model proposed in this study: (**a**) conceptual spectrum to explain structural dynamic properties affected by structural integrity and environments; (**b**) concept of a conventional SHM based on a FE model; (**c**) concept of a SHM based on a FE-based digital twin model.

In this research, hence, we aim to develop a baseline digital twin that comprehensively reflects a part of the environmental changes and their impacts on the structure. Figure 5 presents what this study proposes. Let us assume the variation of the structural integrity and the environmental factors as the blue and red spectrum, respectively, and then, the measured dynamic properties as the pink to purple spectrum as shown in Figure 5a. It means that the measured dynamic properties are affected by both the structural and environmental effects, as mentioned previously. In general, while the environments fluctuate largely and continuously, the structural integrity does not except for disasters or accidents. Therefore, the usual variation of the dynamic properties would be thought to be attributable to environmental change. As shown in Figure 5b, however, it is the traditional way to use only an FE model to focus on the structure itself. That is, the conventional way could reach a conclusion that the change of the dynamic properties arising from the environmental effects in reality is due to the structural state, and thereby, the structural integrity would be easily over- or under-estimated. At every inspection, moreover, the work to find the unclear result should be repeated, such as the model updating as shown in Figure 5b.

On the other hand, an FE-based digital twin model proposed in this research includes the environmental effect as well as the structure itself. Therefore, it can provide the dynamic properties of the structure including the environmental effects, as shown in Figure 5c. To explain, once the environmental circumstance is input to this digital twin model, it outputs the dynamic properties inclusive of the environmental effects. Ultimately, its aim is that the digital twin model can predict the dynamic characteristics in semi-real time if the environmental condition is continuously given. In addition, this merit will be helpful for immediate abnormal detection when its predicted output has a significant difference from the measured dynamic properties in reality under the same environmental condition. Therefore, it can be a proper solution to compensate for the accidental situation. On this point, this study proposed the baseline digital twin model as the starting point of the offshore structure digital twin shown in Figure 5c.

**Figure 6:**
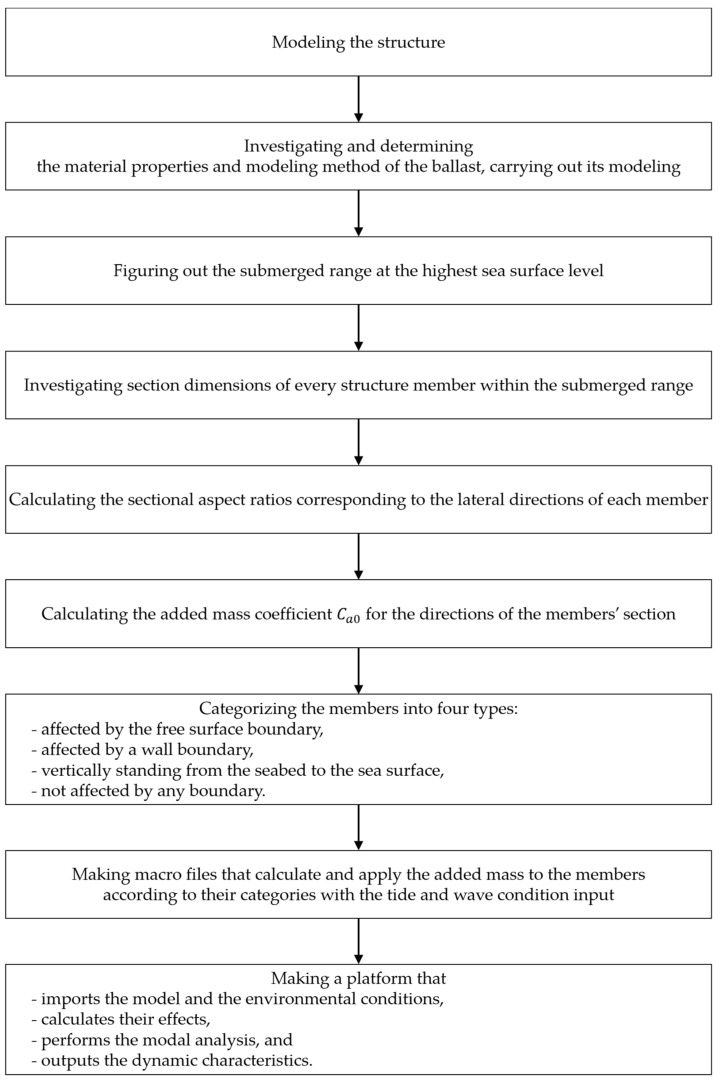
Modeling process of OWC wave power generator structure digital twin.

To achieve this, several steps were undertaken as shown in Figure 6. To summarize, firstly, the simulation model from [6] was thoroughly reviewed to identify disparities between the model and the real structure. Subsequently, necessary modifications were made to the FE model using ANSYS Mechanical APDL to ensure a faithful representation of the actual state of the structure. For instance, we introduced additional modeling for the two different materials in the ballasts. Secondly, we addressed the added mass effect resulting from hydrodynamic phenomena for individual members of the structure, adhering to rules of classes such as Det Norske Veritas (DNV). To achieve this, in depth, all members are categorized into four groups depending on the type of hydrodynamic effect on the members, such as the free surface and wall effect. Lastly, our investigation delved into the dynamic characteristics of the model, along with the influence of the environmental factors considered in the simulation. The insights gained from this research will provide valuable information for future structural assessments and monitoring. Through this work, we aim to develop a digital twin that accurately captures the influence of environmental changes, thereby enabling more effective and informed decision making in managing the structural integrity of the system.

## 2. Materials and Methods

### 2.1. Specification of the OWC Generator Structure

**Figure 7:**
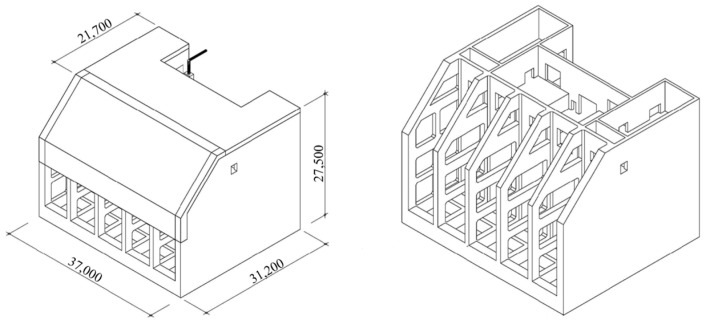
Specification of Yongsu wave power generator structure (unit: mm) [6].

The OWC structure was a fixed-type concrete box structure and the material properties of the reinforcement concrete are shown in Table 2 [6]. The density is 2400 kg/m^3^, elastic modulus and Poisson ratio is 29.8 GPa and 0.18, respectively. The length, width, and height above the seabed were 31.2 m, 37.0 m, and 27.5 m, respectively. The structure has two parts. The fore part is the open chamber room with a slanted roof. The outside sea water is inside, and therefore, the free surface level of the water column rises and falls depending on the outside wave state. The aft part is the caisson which is divided into two compartments. The upper compartment is for the generator system. This space is connected to the chamber room through the holes in the upper wall splitting the fore and aft parts, as presented in Figure 7 [6]. The lower compartment is ballast spaces where the sand and water were filled inside.

### 2.2. Structure

The concrete structure itself was modeled with the elastic modulus and Poisson ratio on the pre-made simulation model [6]. This would be effective because the model was only used for static analysis with a conservative point of view. In this study, however, other things simplified in the preliminary model shall be considered and modeled. The reason for this is that this model was made to mimic its dynamic characteristics for the purpose of applying this model to a digital twin. Theoretically, dynamic characteristics, such as natural frequency, are affected by stiffness, mass, and damping. In general, the stiffness is applied by the modulus of elasticity and Poisson ratio while the mass and damping are applied by the density and the damping ratio of the element materials on the FE model. Therefore, the previous model shall be modified by additionally applying the density and the damping ratio of the reinforcement concrete.

General concrete structures are made of reinforced concrete in which steel bars are buried inside to compensate for less tensile strength of concrete. Regardless, most concrete structures have been modeled under the assumption that the material is not composite but homogeneous. For example, Teughels and Roeck [17] made an FE model of a concrete bridge, and Deng and Cai [18] partially modeled a concrete bridge by applying solid-type elements. As well as those, other several similar studies [19,20] carrying out the modelling of concrete structures thought the concrete material was homogeneous. Therefore, this study also applied the material properties of concrete as shown in Table 2.

This study models the wave power generator structure by using ANSYS Mechanical APDL [21]. Referring to the pre-made simulation model in Figure 4a [6], an FE model of the concrete structure is made up of three different element types, beam, shell, and solid element types, as shown in Figure 4b. The beam elements are green whereas the shell elements are cyan. Assuming that the bottom of this structure is rigidly connected to the seabed, all nodes on the surface were fixed in the six degrees of freedom as the boundary condition.

### 2.3. Ballasts

**Figure 8:**
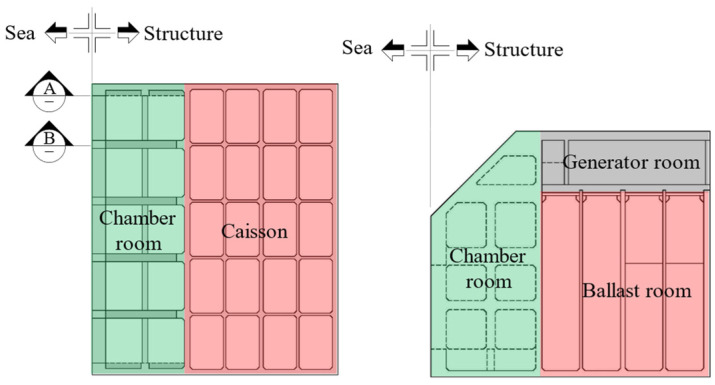
Plan and section outline with the view A and B [6].

**Figure 9:**
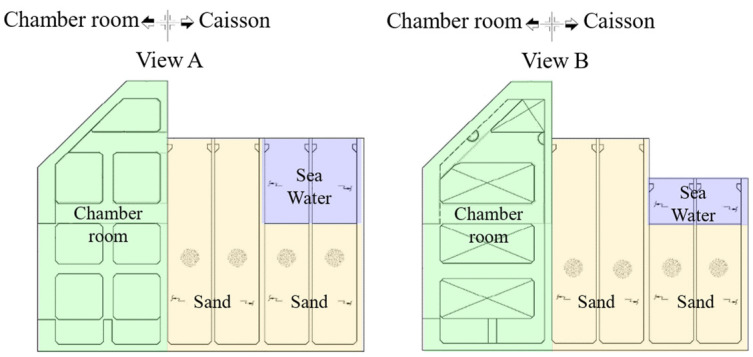
Section view A and B [6].

In addition to the structure itself, others influencing the structural dynamic properties exist but were not considered in the pre-made model. They are the ballast, added mass, and hydrodynamic damping. As for the ballast, at first, the caisson is separated into a total of twenty spaces on plane view by four along with the length and five along with the width as shown in Figure 8. To explain, among the twenty spaces, ten spaces closer to the chamber room are called the fore-ballast part, and others are called the aft-ballast part. As shown in Figure 9, the sand and sea water are filled in the ballast room of the caisson. The eight spaces out of the fore-ballast part are filled with sand, except two middle spaces. The aft-ballast part is filled with sand and sea water.

As for the two materials, sea water and sand, contained in the caisson, density, elastic modulus, and Poisson ratio, which are the material properties affecting the structural dynamic characteristics, are surveyed. Sand is known as fairly uncertain in mechanical properties. This is because, in general, sand is a stack of various soil grains. It means that the size of sand particles and the amount of moisture content and pressure in the sand are not definite. Table 3 summarized representative research results on the properties of sand, and non-negligible distinction is noticeable depending on the type of sand.

Considering the fact that the sand in the ballast space was just dropped into the caisson with to the intention of filling the structure, the material properties of the sand were applied as shown in Table 4 in this study. Table 4 also shows the properties of sea water. The modulus of elasticity is considered to be 2.07 × 10^3^ MPa [24]. Note that the Poisson ratio is 0.5 in reality but 0.49 was applied for stable numerical analysis.

**Figure 10:**
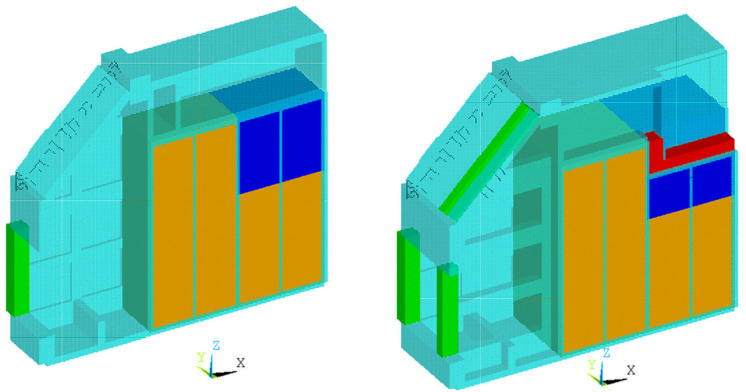
Sand (brown) and sea water (blue) in ballast spaces (section view-A **left**; B: **right**).

As shown in Figure 9, most volumes of the caisson are filled by sand and sea water, which means that how to apply those in the model is important. Depending on the purpose, there are two approaches to modeling such material that is filled inside a structure. For static- or quasi-static analysis, it is usually modeled as a force, i.e., just a part of self-weight or inertia force, and therefore, the density of the adjacent structural members is increased or nodal mass elements are added to the members. The ballast of a ship or an offshore structure is a general example of that method. For dynamic analysis like modal analysis, on the other hand, there is one referable research example. To make an FEM model and investigate the dynamic properties of a dam structure, ref. [24] modeled the water contained in a dam as a solid-type element. Therefore, the latter way is used to model the sand and sea water since the aim is matched with this study as shown in Figure 10.

### 2.4. Hydrodynamic Effects

Hydrodynamic effects, such as added mass and hydrodynamic damping, shall be considered. Above all, the added mass is the mass of fluid surrounding an object, and therefore accelerated together with the object when the object is accelerated. The amount of the fluid mass that is accelerated may be calculated by the potential theory if a simple object like a circular cylinder or a sphere is submerged under deep water relative to the size of the object. In the case of a complicated shape, on the other hand, it is difficult to compute the added mass theoretically, and therefore, the experimental approach would be applied. Next, hydrodynamic damping is another factor to be considered. In general, two approaches can be used to apply damping: an empirical approach is suitable for relatively slender structures, while a theoretical approach is necessary for larger structures. In addition, the two factors, i.e., the added mass and hydrodynamic damping, are impacted by the boundary, which is the fluid domain where an object is submerged, and the size and location of the object, as well. As a result, determining the exact amount of hydrodynamic added mass and damping can be challenging. To quantitatively calculate those effects for complex structures like this problem, there would be two possible ways. The first is to find numerical solutions by using computational fluid dynamic (CFD) techniques. The second is to find theoretical solutions by using the traditional potential theory, and there are several commercial hydrodynamic programs, such as WAMIT and ANSYS AQWA. These are known to basically use a hydro panel model. However, this study does not extend this matter to those solutions and leaves them for the scope of future study. Instead, a more intuitive and empirical approach is applied to establish the baseline digital twin model effectively in the beginning.

#### 2.4.1. Approach

It is very hard to precisely identify the hydrodynamic effects, for example, the added mass and hydro damping of a huge and complicated structure in the real world, through mathematical theories or experiments. For this reason, approximate methods based on relevant acknowledged studies that have been conducted and accumulated by many outstanding scholars for a long time are summarized and provided by classes such as Det Norske Veritas (DNV), American Bureau Veritas (ABS), and Lloyd Register (LR). Since it is reliable and useful for the practical application of the matter, lots of engineers around the world widely utilize those, and therefore, this study also applies them, particularly referring to DNV-RP-C205 [25].

Added mass of a structure is determined by its section and direction of motion, essentially when it is far from the fluid domain boundary. In detail, it is parameterized by two factors: first, the reference area ($\pi a^{2}$), which is the area of the circle with a diameter equal to the projected length of the section for the direction of motion (known as the specific radius a), and second, the aspect ratio, which is the ratio of a for b, the half of another section length. This means that even a rectangular section can be simplified as a circular area to calculate its added mass, and each direction must be evaluated independently. The Keulegan–Carpenter number (*KC* number) and surface roughness of the member also affect the added mass. As shown in Equation (1), the *KC* number is explicitly expressed as the product of the period T and the amplitude $v_{m}$ of the fluid velocity variation over the (specific) diameter D of the structure, and connotes the relation of the drag force and inertia force. If the *KC* number is less than 3, not only the *KC* number but the roughness does not affect the added mass, and thereby, the added mass coefficient $C_{a}$ = 1. Conversely, if it is larger than 3, $C_{a}$ decreases as the *KC* number increases, and the rougher it is, the lower as shown in Equations (2) and (3). Here, Δ is the surface roughness, and $C_{DS}$ is the drag coefficient depending on the roughness in a steady flow.

(1) $$KC=\frac{v_{m}T}{D},$$

(2) $$C_{a}=\max\left\{\begin{array}{c} 1.0-0.044(KC-3)\\ 0.6-(C_{DS}-0.65) \end{array}\right.,$$

(3) $$C_{DS}=\left\{\begin{array}{c} \Delta <10^{-4}\;\left(smooth\right):0.65\\ 10^{-4}<\Delta <10^{-2}\;:\frac{29+4\log_{10}\Delta }{20}\\ 10^{-2}<\Delta \;\left(rough\right):1.05 \end{array}\right.,$$

Regarding a structure close to the boundary of the fluid domain, meanwhile, the boundary effect shall be carefully considered. The free surface or wall interaction is a representative example of the boundary effect. DNV-RP-C205 mainly refers to the research on a circular cylinder by Greenhow and Yanbao [26] and Greenhow and Ahn [27]. According to those references, the radius of section, r, and the distance to its center from the boundary, H, are major factors of the boundary effect. At first, the added mass coefficient $C_{a}$ close to a wall is determined by Equations (4) and (5). Here, H<r means the cylinder is not able to move to the wall but able to be parallel to the wall whereas H>r means the cylinder can move in both directions. In the case of H=r, Equation (4) diverges to infinity, and thereby, $C_{a}=\frac{\pi^{2}}{3}-1$ from analytic derivation in that case. In fact, that value is the largest peak value. Note that the added mass coefficient $C_{a}$ nearly becomes converged to 1 when H is four times r. In other words, the wall boundary effect for the added mass almost dissipates when the wall is twice as far away as the diameter of the structure.

(4) $$C_{a}=\left(2\cosh\left(2s_{0}\right)-2\right)\left(\frac{1}{12}+\frac{\pi^{2}}{12s_{0}^{2}}-\frac{1}{2s_{0}}\right)-1,$$

(5) $$s_{0}=\ln\left(\frac{H}{r}+\sqrt{{\left(\frac{H}{r}\right)}^{2}-1\;}\right),$$

Second, the added mass coefficient $C_{a}$ near the free water surface is also dependent upon the distance between the structure and the boundary. Unlike the wall effect, it tends to vary monotonically without a peak according to the distance. In contrast to the wall effect, in addition, the cylinder is allowed to move in both parallel and perpendicular directions to the free surface. Importantly, the added mass coefficients are different from each other when H<r. The added mass coefficient in the direction normal to the free surface is presented in Equations (6) and (7). The value in the direction parallel to the free water surface was approximated from the graph in the literature [26,27]. When H=r, the added mass coefficient $C_{a}=\frac{\pi^{2}}{6}-1$ is derived by the analytic solution. When H>r, on the other hand, the added mass coefficients are the same as each other. As H increases, meaning that the cylinder moves farther from the water surface, the added mass coefficient also increases and converges to 1 when the structure is far, specifically, about four times the structure radius from the free surface, as it does for the wall effect.

(6) $$C_{a}=\left\{\begin{array}{c} \frac{\pi^{2}}{3}\frac{1-\cos s_{0}}{{\left(2\pi -s_{0}\right)}^{2}}+\frac{1-\cos s_{0}}{6}+\frac{\sin s_{0}-s_{0}}{2\pi }\;(-r<H<r)\\ \frac{\pi^{2}}{6}-1\;(H=r) \end{array}\right.,$$

(7) $$s_{0}=2\cos^{-1}\left(-\frac{H}{r}\right)(-r<H<r),$$

Third, [25] suggests the added mass coefficient of a circular cylinder vertically standing from the seabed to above the free water surface as shown in Equation (8). Here, k and r is wave number and radius of the cylinder, respectively. $A_{1}$ is derived from the derivative of Bessel functions of first order as shown in Equation (9). Thus, $C_{a}\to 1$ when kr→1, i.e., the wave length becomes longer. In detail, this equation can be derived for the specific case of a circular cylinder from the McCamy–Fuchs correction [28], a widely used method for applying diffraction effects to hydraulic inertial forces.

(8) $$C_{a}=\frac{4}{\pi {\left(kr\right)}^{2}\sqrt{A_{1}(kr)}}-1,$$

(9) $$A_{1}\left(kr\right)=J_{1}^{\prime 2}\left(kr\right)+Y_{1}^{\prime 2}\left(kr\right),$$

Rahman and Bhatta [29] further studied the vertical cylinder. Specifically, the horizontal hydrodynamic forces of the diffraction waves from the cylinder were defined directly from the potential flow theory, and the added mass was derived by the sum of those forces. The total dimensionless added mass is expressed as shown in Equation (10). Here, μ is the total added mass of the cylinder, respectively, $\rho_{F}$ is the fluid density, d is the depth, $K_{1}$ is the modified Bessel functions of second order, and, $H_{1}^{1}$ is the Bessel functions of third order (i.e., Henkel function). k is the wave number when $k^{2}>0$, $k_{n}$ is the n-th wave number when $k^{2}<0$, Re means the real part, $d_{0}$ and $d_{n}$ in those equations is as shown in Equation (11), and $D_{0}$ and $D_{n}$, the hyper parameters of $d_{0}$ and $d_{n}$, are as shown in Equation (12).

(10) $$\frac{\mu }{\rho_{F}r^{3}}=-\pi \left[\frac{kd_{0}^{2}}{{\left(kr\right)}^{2}}Re\left(\frac{H_{1}^{1}\left(kr\right)}{{H_{1}^{1}}^{\prime }\left(kr\right)}\right)+\sum_{n=1}^{\infty }\left\{\frac{k_{n}d_{n}^{2}}{{\left(k_{n}r\right)}^{2}}\frac{K_{1}\left(k_{n}r\right)}{K_{1}^{\prime }\left(k_{n}r\right)}\right\}\right],$$

(11) $$d_{0}=\frac{\sinh kd}{kD_{0}},\;\;\;d_{n}=\frac{\sinh k_{n}d}{k_{n}D_{n}},$$

(12) $$D_{0}=\sqrt{\frac{d}{2}\left(1+\frac{\sinh2kd}{2kd}\right)},\;\;\;D_{n}=\sqrt{\frac{d}{2}\left(1+\frac{\sinh2k_{n}d}{2k_{n}d}\right)},$$

On top of added mass, hydrodynamic damping is another important factor to con-sider for the motion of an object in the water. However, accurately determining the damping coefficient is a complex matter as it is influenced by various factors, including the shape and surface roughness of the object. Furthermore, the method for estimating damping is significantly dependent on the relationship between the member size and fluid particle velocity. Currently, the fluid particle velocity in the chamber room is unknown, and therefore, further study is required to address this issue.

#### 2.4.2. Site Condition

**Figure 11:**
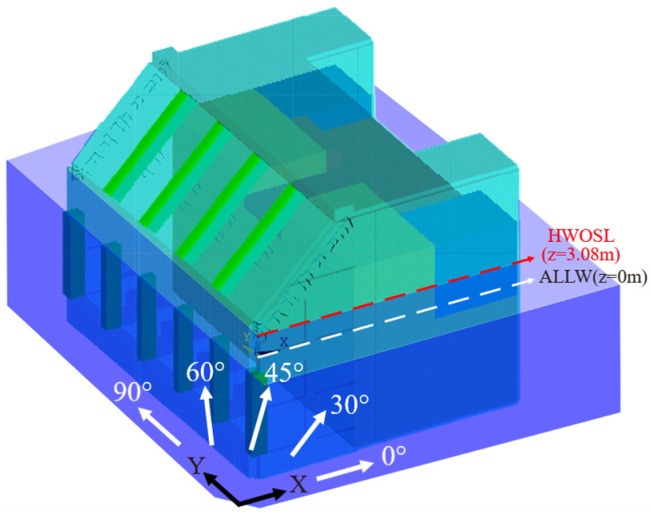
Water depth and wave directions for the structure.

The hydrodynamic effects of each structural member must be modeled, taking into consideration their specifications and characteristics for fluid boundaries, such as the water surface and seabed. Hence, the sea surface level and depth must be well defined. Generally, the sea surface rises and falls due to tides. Figure 11 displays the tide range from the approximate lowest low water level (ALLW), which is the datum level (z = 0) in Korea, to the highest water ordinary spring tide level (HWOSL) of 3.08 m, which was determined by tidal harmonic analysis. To apply the hydrodynamic effects to a digital twin model, therefore, this study modeled the hydrodynamic effects to vary in the tide range. To be specific, this study modeled nodal mass elements over the submerged range up to HWOSL from the seabed. Once the depth value and wave condition are provided, the added mass corresponding to those conditions is distributed equally over the given depth range for individual members. In addition, five different wave directions were simulated to investigate the effect of wave conditions on the dynamic characteristics of the structure as shown in Figure 11.

#### 2.4.3. Modeling of Added Mass Effect

**Figure 12:**
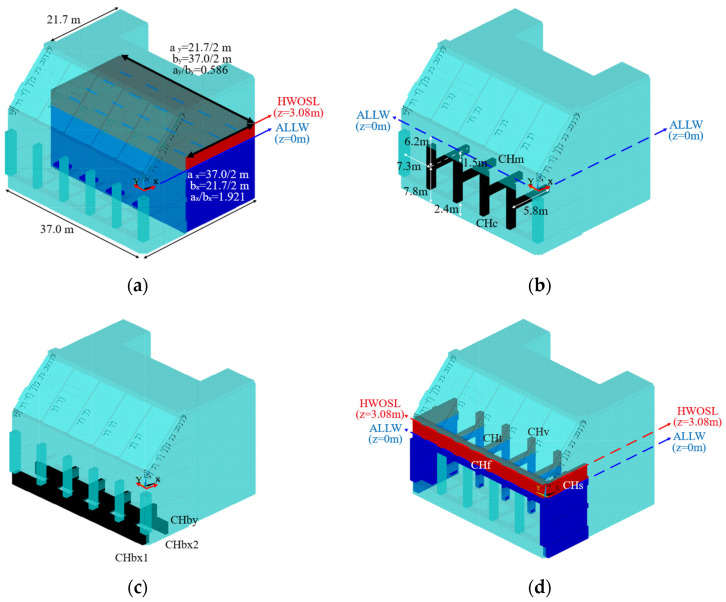
Four types of members for the hydrodynamic effects: (**a**) caisson; (**b**) members far from the fluid boundary; (**c**) members on seabed; (**d**) members affected by the free surface.

Based on the water surface level shown in Figure 11, the structure members are categorized for hydrodynamic effect, as displayed in Figure 12. Some members, as shown in Figure 12a, are far from the fluid boundaries considering their section size, while others, as shown in Figure 12b–d, are not, and therefore are affected by the free water surface or the fixed boundary, such as the seabed.

To begin with, the added mass coefficient of the reference area $C_{a0}$ for individual directions that the section of a member is thought to be allowed to move is obtained based on the aspect ratio of the rectangle without any considerations, such as the boundary effects. Subsequently, if such effects validly occur on the member considering its characteristics, such as the location and direction in the water, those effects are applied to $C_{a0}$, and finally, result in the determination of $C_{a}$.

For instance, the caisson in Figure 12a can be viewed as a rectangle section column, and $C_{a0}$ corresponding to its aspect ratios for the X and Y direction is primarily found in Table 5. It is vertically penetrating from the seabed to the free surface, making itself appropriate to evaluate the hydrodynamic effect according to [29]. Therefore, $C_{a}$ is calculated by applying Equations (10)–(12) to $C_{a0}$. It is noted that the effect is embodied by relying on the free surface elevation $z_{t}$ in this study. 

CHm and CHc in Figure 12b are member groups located in the middle of the chamber room, with CHm being horizontal members and CHc being vertical members. These members are assumed to be capable of moving in two perpendicular directions to their length. Specifically, CHm is assumed to be able to move in the Y and Z directions, while CHc is assumed to move in the Y and X directions, respectively. Table 6 provides information on the section dimensions, the specific radius (a), the aspect ratio (a/b), and the added mass coefficient ($C_{a0}$) for each direction. It is worth noting that for each direction, H/r, which denotes the distance from the section center to the boundary relative to the radius, is significantly greater than 4 under the assumption of the radius r being equal to the specific radius a. Thus, it is convincing that these members are far enough not to affect the fluid boundary, and vice versa, and therefore, $C_{a0}$ can be used as the added mass coefficient without any modification for the boundary effect.

CHbx1, CHbx2, and CHby in Figure 12c are member groups located on the bottom of the chamber room. These members are assumed to be unable to move in Z direction because they are fixed at the bottom, and also assumed to be able to move a horizontal direction perpendicular to their length. Therefore, CHbx1 and CHbx2 are assumed to be able to move in the X direction, and CHby is assumed to move in the Y direction, respectively. Table 7 lists the section dimensions, the specific radius (a), the aspect ratio (a/b), and the added mass coefficient ($C_{a0}$) for each direction. Because of H=r for these group members in Equation (4), $C_{a0}$ shall be modified to apply the wall effect as $C_{a}$ in Table 7. 

The structure members categorized as CHt, CHv, CHf, and CHs in Figure 12d are placed within the tide range, and their added mass relies on the free surface elevation $z_{t}$. Therefore, this study considers not only the free surface effect but the characteristics of the individual member when determining the added mass. As shown in Figure 12d, the blue area below ALLW (z = 0 m) is always submerged, and the added mass always arises, as well. Conversely, the red area between ALLW and HWOSL (z = 3.08 m) may not be submerged based on the sea surface elevation, and therefore, the added mass is applied depending on the $z_{t}$ in this study.

In detail, the horizontal members of CHt near the free surface range are assumed to be able to move in two horizontal directions X and Y, and the added mass for each direction is shown in Table 8. The members are partially submerged when the tide is at ALLW and completely submerged when the tide is higher. This study applies the phenomenon according to Equations (6) and (7).

In the case of CHv, the four vertical columns attached to the caisson, the effect of added mass on the Y-axis motion is dominant due to their behavioral characteristics, so the basic added mass coefficient in that direction is shown in Table 8. Since the members are attached to the wall, $\frac{\pi^{2}}{3}-1$ was applied to $C_{a0}$, assuming that H=r. For the range that is out of the water depending on the tide level, the added mass was considered corresponding to the tide.

For CHf, which is an extension of the chamber room ceiling and acts as the front upper vertical wall, the main consideration is the effect of added mass on the X-axis behavior. Unlike CHt, CHf is not fully submerged in water even at HWOSL, and hence, a free water surface effect of $\frac{\pi^{2}}{6}-1$ was applied to $C_{a0}$ for the section profile from its bottom to the waterline, assuming H=r.

The side walls CHs of the chamber are basically the shape of a plate, which is expected to have the added mass effect on the dominant behavior in the Y-axis direction. To explain, the aspect ratio a/b is greater than 10, which is regarded as a flat plate. Therefore, $C_{a0}$ of 1 in this direction would be reasonably obtained. In addition, these two walls stand from the seabed like the caisson, and therefore, the added mass calculation presented in Equations (8)–(10) are considered together to match the input tide level.

## 3. Results and Discussion

It is difficult to visually verify the modeling results. Therefore, a comparative analysis is performed on the total mass, natural frequencies, and mode shapes calculated from the preliminary model in the reference review report (Model R), the model with ballasts (Model 1), and the model where the added mass is applied to Model 1 (Model 2). 

Table 9 presents the masses of Model R and Model 1. The reference model (Model R) has a mass of 19,975 tons, while the mass of Model 1 is 41,673 tons. This indicates that the ballast materials have a mass of approximately 21,698 tons, exceeding the mass of the structure.

**Figure 13:**
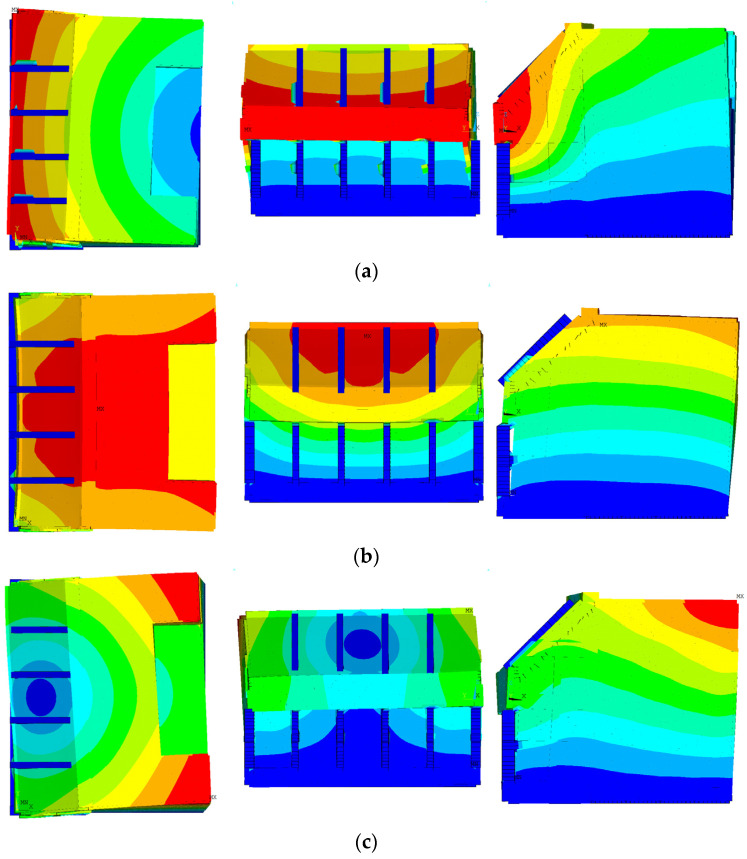
Mode shape of Model R: (**a**) first mode (7.537 Hz); (**b**) second mode (8.095 Hz); (**c**) third mode (12.354 Hz).

Figure 13 illustrates the first three mode shapes of Model R. Figure 13a shows the lateral response of the structure in the first mode, with a natural frequency of 7.537 Hz. This is reasonable since there are no structural members capable of bearing force in the Y-direction in the chamber room. Figure 13b displays the second mode shape, which occurs in another horizontal direction at a frequency of 8.095 Hz. On the other hand, the third mode involves torsional behavior along the Z-axis, with a relatively higher natural frequency of 12.354 Hz compared to the two lower modes. It is common that the whole structure moves in the lowest three mode shapes.

**Figure 14:**
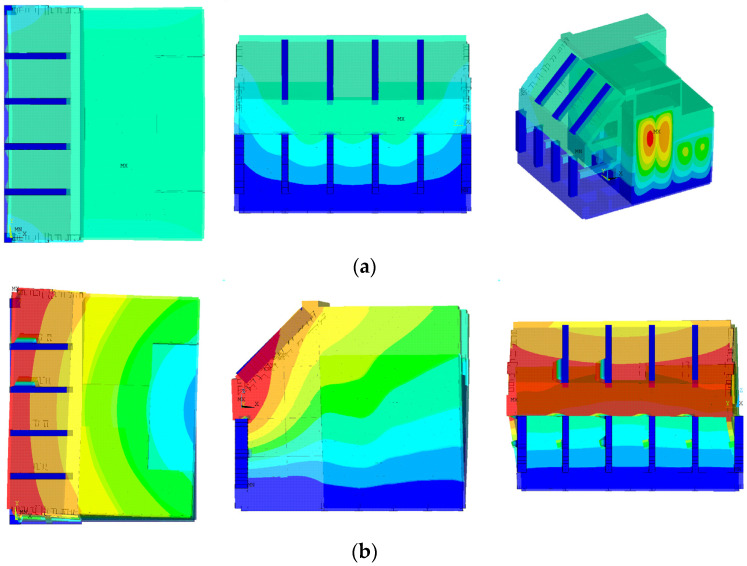
Mode shape of Model 1: (**a**) first mode (5.694 Hz); (**b**) second mode (5.976 Hz).

However, the mode shapes of Model 1 differ from those of Model R, as illustrated in Figure 14. Figure 14a displays the first mode shape, indicating that a specific portion of the ballast space responds exclusively at the lowest natural frequency of 5.694 Hz. This discrepancy arises due to the presence of a significantly less stiff material, such as sand, filling the ballast space, in contrast to the concrete structure. The global behavior corresponding to the first mode shape of Model R occurs in the second mode, with a natural frequency of 5.976 Hz. Moreover, other global mode shapes exactly corresponding to the second and third mode of Model R do not transpire, because many local mode shapes like Figure 14a and mixed-mode shapes coupled with global and local behaviors are identified in higher modes.

Model 2 incorporates the added mass in addition to Model 1. The added mass is calculated based on the design wave and tidal conditions provided in Table 10, with the tidal level set to the HWOSL condition. It is noted that the current speed is not considered in this study.

**Figure 15:**
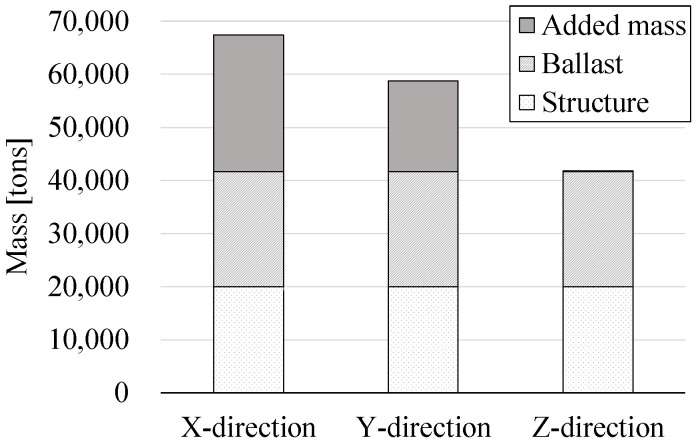
Mass components of Model 2.

Figure 15 illustrates the amounts of each mass component in Model 2 for different directions under these ocean environmental conditions. As depicted in Figure 7, the caisson’s specific diameter is larger in the X-direction compared to the Y-direction, resulting in a greater added mass in the X-direction. Conversely, the added mass in the Z-direction is only small since it is limitedly applied to certain small horizontal members within the chamber room.

Similar to Model 1, the global mode shape corresponding to the first mode of Model R happens as the second mode, and that lateral mode shape is the only common global mode occurring in all three models. For this reason, the natural frequencies of that mode are enumerated for comparison in Table 11. Compared to the natural frequency of Model R, approximately 20.7% of the natural frequency decreased, resulting from the ballast in the caisson structure. About 7.90% is further lowered due to the added mass effect in Model 2. This result clarifies the necessity of modeling the ballast components and hydrodynamic mass effect in using the dynamic characteristics of these types of offshore structures.

Regarding Model 2, it is possible to investigate the influence of tide levels on the dynamic properties. The natural frequency of the lowest global mode is simulated for four different tide cases, ranging from HWOSL (3.08 m) to LLW (0 m) by 1 m, as presented in Table 12. For this structure, as the tide level decreases, the natural frequency of that mode also decreases. This can be attributed to the reduction in added mass corresponding to the tide level. It emphasizes the importance of considering such an effect from this perspective.

Moreover, Table 13 listed the varying natural frequencies of that global mode depending on the incident wave direction at HWOSL shown in Figure 11. The natural frequency reaches the largest amount when the waves approach the lateral side of the structure. This variation is attributed to the added mass coefficient and specific radius of the structure in different directions.

Table 14 presents the projected range of variation in natural frequencies resulting from environmental changes. Our simulations indicate that the natural frequency is expected to fluctuate by a maximum of 4.81%. To provide a real-world reference, we refer to the work of Yi et al. [10], who conducted a study on the Gageocho Ocean Research Station (ORS), an offshore structure of the jacket type. In their investigation, they observed that the natural frequency of the ORS’s first lateral bending mode fluctuated within a range of approximately 1.23%. Thus, it is evident that the natural frequencies of the concrete wave power generator structure we studied are expected to exhibit variations four times larger than those observed in the actual jacket-type offshore structure.

Furthermore, in a study conducted by Kim et al. [5], it was found that the impact of joint damage on the natural frequencies of the ORS was significantly smaller compared to the measured variation range of 1.23% on site reported by Yi et al. [10], and it was pointed out that this influence may be an obstacle to assuring the accuracy of damage detection. Consequently, it can be inferred that, for box-type concrete structures like the one studied here, the environmental effects are required to be embodied much more precisely. This is because the natural variation arising from the ocean environment is substantially greater in this structure compared to the variation observed in the ORS.

Therefore, this study needs to be further developed in two different directions. One is to model the environmental effect elaborately by utilizing the numerical or theoretical solution. Another is to conduct the parameter tuning explained in Figure 5c. To do so, some preliminary studies need to be carried out. The representative area shows how to design and construct the measurement system, such as the measurement points, and there are some latest references for an optimal layout of sensors [30,31]. Also, the errors between the measured data and the digital twin model need to be investigated for accurate parameter tuning.

## 4. Conclusions

In this study, a finite element model was developed to create a digital twin of a wave power generator structure operating off the coast of Yongsu-ri, Jeju-do, South Korea. The digital twin incorporates non-structural mass components, specifically the ballast and added mass surrounding the structure, resulting in a more accurate and realistic model. The ballast component was represented using hexahedral elements with physical properties tailored to individual materials, while the added mass was modeled as a mass element at all nodes in contact with sea water. The degree of added mass was determined using a widely accepted method employed by classification societies, accounting for the influence of environmental factors such as tide level and wave direction.

The developed digital twin was capable of providing dynamic characteristics of the structure, encompassing both the ballast masses and the added mass corresponding to varying tide and wave conditions. Our findings underscore the importance of considering non-structural mass components to accurately capture the dynamic behavior of the structure. Furthermore, we observed that environmental factors, such as tide level and wave direction, exerted a more pronounced influence on the dynamic characteristics of the box-type structure compared to lightweight offshore structures like jacket-type structures.

For future research, in terms of enhancing the accuracy of the baseline digital twin model, we propose to employ added mass and fluid damping values calculated based on fluid dynamic theory rather than empirical methods. Additionally, simulating more realistic boundary conditions can be achieved by implementing frictional contact at the bottom surface of the caisson on the seabed. These improvements will contribute to refining the predictive capabilities of the digital twin, enabling more effective structural assessments and monitoring of the wave power generator in real-world marine environments. Furthermore, when it comes to applying this digital twin model to the real structure, the optimal measurement plan for the real structure needs to be established. Once the measured data are gathered, as shown in Figure 5c, overall parameter tuning on the uncertain factors, such as the mechanical properties of the materials and hydrodynamic coefficients, shall be carried out to completely fit this model to reality.

## Figures and Tables

**Table 1:** General causes of disparity between a simulation model and a real structure.

Type	Cause	Aspect	Reason	Alternative Solution
General structure	Simplification	Model	For conservative and convenient modeling, 1D/2D element types are used to model a real structure in general, and minor structural members are often ignored.	-
	Non-structural mass omission	Model	For a conservative safety evaluation, mass components, such as appurtenance, equipment, and facilities, are usually regarded as the weight or density increase.	Mass modeling and Initial tuning
	Boundary condition	Model	The boundary condition is frequently simplified to the six degrees of freedom.	Advanced modeling and Initial tuning
		Reality	Civil foundations, such as piling and grouting, are affected by the uncertainty of geotechnical property and interaction.	
	Material uncertainty	Reality	Concrete materials entirely depend on the curing completeness.	Initial tuning
	Construction error	Reality	Sizes and spacings of rebars in reinforcement concrete have uncertainty in the construction process.	-
Offshore structure	Biofouling	Model	For a conservative safety evaluation, the thickest biofouling is considered as well as frequently modeled as the weight load instead of its volume having mass.	Parametric modeling
		Reality	The thickness, range, density, and roughness of the biofouling are not clearly figured out in reality because they vary over time.	Varying over time
	Corrosion	Model	For a conservative safety evaluation, in general, the maximum corroded member sections are assumed and modeled.	Parametric modeling
		Reality	The degree and range of the corrosion are difficult to clarify in reality.	Varying over time
	Hydrodynamic effects	Model	For a conservative safety evaluation, the hydrodynamic effects are usually regarded as the forces, not as the mass.	Parametric modeling
		Reality	The degree of the hydrodynamic effects, such as the added mass or fluid damping effect, relies on the environmental conditions.	Varying over time

**Table 2:** Material properties of the reinforced concrete [6].

Density	Elastic Modulus	Poisson Ratio
2400 kg/m^3^	29.8 × 10^3^ MPa	0.18

**Table 3:** Mechanical properties relying on the type of sand.

Reference	Type of Sand	Elastic Modulus (MPa)	Poisson Ratio
Das [22]	Loose	10.35–24.15	0.20–0.40
	Medium dense	17.25–27.60	0.25–0.40
	Dense	34.50–55.20	0.30–0.45
	Silty	10.35–17.25	0.20–0.40
	Sand with gravel	69.00–172.50	0.15–0.35
Bowles [23]	Loose	10–25	0.3–0.4 (commonly used)
	Dense	50–81	
	Silty	5–20	
	Sand with gravel	50–200	

**Table 4:** Material properties of sand and sea water.

Material	Density	Elastic Modulus	Poisson Ratio
Sand	1835 kg/m^3^	10.0 MPa	0.30
Water	1024 kg/m^3^	2.07 × 10^3^ MPa	0.49

**Table 5:** Added mass coefficient of the caisson structure.

Member	Section (m)		Direction	*a* (m)	*b* (m)	Aspect Ratio	$C_{a0}$	$C_{a}$
	Width	Length				(*a*/*b*)		
Caisson	37.0	21.7	X	18.50	10.85	1.92	1.404	$z_{t}$ *
			Y	10.85	18.50	0.57	1.667	$z_{t}$ *

* $z_{t}$ means the value determined depending on $z_{t}$.

**Table 6:** Added mass coefficient of the member group CHm and CHc.

Member	Section (m)		Direction	*a* (m)	*b* (m)	Aspect Ratio	$C_{a0}$	$C_{a}$
	Width	Length				(*a*/*b*)		
CHm	1.00	1.50	Y	0.75	0.50	1.50	1.435	9.73
			Z	0.50	0.75	0.67	1.637	12.4
CHc	1.00	1.70	Y	0.85	0.50	1.70	1.405	8.59
			X	0.50	0.85	0.59	1.666	11.6

**Table 7:** Added mass coefficient of the member groups CHbx1, CHbx2, and CHby.

Member	Section (m)		Direction	*a* (m)	*b* (m)	Aspect Ratio	$C_{a0}$	$C_{a}$
	Width	Length				(*a*/*b*)		
CHbx1	1.70	2.40	X	1.20	0.85	1.50	1.448	3.316
CHbx2	0.80	2.40	X	1.20	0.40	3.00	1.310	3.000
CHby	1.00	2.40	Y	1.20	0.50	2.40	1.340	3.068

**Table 8:** Added mass coefficient of the member groups CHt, CHv, CHf, and CHs.

Member	Section (m)		Direction	*a* (m)	*b* (m)	Aspect Ratio	$C_{a0}$	H/r
	Width	Length				(*a*/*b*)		
CHt	1.00	2.00	Y	1.00	0.50	2.00	1.36	$z_{t}$ *
			Z	0.50	1.00	0.50	1.70	$z_{t}$ *
CHv	1.00	1.35	Y	0.68	0.50	1.36	1.46	1
CHf	1.70	1.50 + $z_{t}$	X	1.50 + $z_{t}$	1.70	$z_{t}$ *	$z_{t}$ *	1
CHs	1.04	1.15	Y	5.58	0.52	10.73	1	-

* $z_{t}$ means the value determined depending on $z_{t}$.

**Table 9:** Total mass of Model R and Model 1 (tons).

Component	Model R	Model 1
Structure	19,975	19,975
Ballast	-	21,698
Total	19,975	41,673

**Table 10:** Ocean environmental condition.

Depth	Tide	Wave			
		Direction	Height	Period	Length
ALLW	HWOSL	0°	8.2 m	12.9 s	161.06 m

**Table 11:** Natural frequency of the lowest global mode in common with three models.

Model R	Model 1	Model 2
7.537 Hz	5.976 Hz	5.514 Hz

**Table 12:** Natural frequency of the lowest global mode depending on tide.

3.08 m (HWOSL)	2 m	1 m	0 m (ALLW)
5.514 Hz	5.574 Hz	5.664 Hz	5.739 Hz

**Table 13:** Natural frequency of the lowest global mode depending on wave directions.

0°	30°	45°	60°	90°
5.514 Hz	5.530 Hz	5.539 Hz	5.547 Hz	5.554 Hz

**Table 14:** Projected range of variation in natural frequency resulting from environmental changes.

Environmental Change	Tide	Wave Direction	Total
Max (A)	5.739 Hz	5.554 Hz	-
Min (B)	5.514 Hz	5.514 Hz	-
Difference (C = A − B)	0.225 Hz	0.040 Hz	-
% (100 × C/B)	4.08	0.73	4.81

## Data Availability

Data are contained within the article.
